# Peripheral blood telomerase activity and telomerase reverse transcriptase gene expression in psychiatric disorders: a systematic review and meta-analysis

**DOI:** 10.1192/j.eurpsy.2025.10099

**Published:** 2025-09-01

**Authors:** Johanna Thurin, Cynthia Marie-Claire, Bruno Etain

**Affiliations:** 1 Optimisation Thérapeutique en Neuropharmacologie, Université Paris Cité, INSERM UMRS 1144, Paris, France; 2 Département de Psychiatrie et de Médecine Addictologique, Hôpitaux Lariboisière-Fernand Widal, Université Paris Cité, Paris, France; 3 Fondation Fondamental, Créteil, France

**Keywords:** cellular aging, psychiatric disorder, telomerase, telomerase activity, TERT expression

## Abstract

**Background:**

Telomere shortening is shared by all psychiatric disorders and is hypothesized as resulting from decreased telomerase activity (TA) or expression of the *TERT* (Telomerase Reverse Transcriptase) gene.

**Methods:**

A search in four English databases was conducted from inception to November 2024 to evaluate the association between psychiatric disorders and telomerase activity (TA) or *TERT* gene expression in peripheral blood. We performed two separate meta-analyses to generate pooled effect size (ES) for TA and *TERT* gene expression, followed by meta-regression.

**Results:**

The systematic review included 16 studies, 14 of which were included in the meta-analyses. When considering all psychiatric disorders, no associations were found for TA (ES = 0.08 [−0.50–0.67], *p* = 0.78 – *I*-squared = 95%), nor *TERT* gene expression (ES = 0.00 [−0.56–0.57], *p* = 0.99 – *I*-squared = 91%). However, TA was elevated in mood disorders (ES = 0.61 [0.06–1.16] – *p* = 0.03), while decreased in non-mood disorders (ES = −0.70 [−1.37 – −0.03] – *p* = 0.04). ES for TA were larger in mood disorders as compared to other disorders (*p* = 0.003).

**Conclusions:**

This meta-analysis shows that psychiatric disorders – taken together – are not associated with peripheral blood TA or *TERT* gene expression. Nevertheless, we find that TA is increased in depressive disorders (unipolar or bipolar), whereas decreased in non-mood psychiatric disorders. The paucity of studies and small sample sizes are important limitations, especially for *TERT* gene expression. Further research is needed, incorporating a broader spectrum of psychiatric disorders and larger sample sizes.

## Introduction

Psychiatric disorders are among the top ten leading causes of burden worldwide [1]. Individuals diagnosed with psychiatric disorders have 14.66 years-of-potential-life-lost (YPLL) compared to the general population [2]. While unnatural causes of death (suicide and drug intoxications) [3] contribute to this premature mortality, psychiatric disorders are also linked to a higher mortality rate due to natural causes such as cardiovascular diseases, diabetes, or cancers [4]. These conditions are recognized as age-related diseases that reflect accelerated cellular aging [5]. Twelve biological processes are identified as hallmarks of cellular aging including telomere attrition, mitochondrial dysfunction, cellular senescence, chronic inflammation or stem cell exhaustion [6].

Telomeres are non-coding structures located at the ends of chromosomes that gradually shorten with cell division [6]. The Hayflick limit refers to the length at which, after repeated cell divisions, telomeres become too short and initiate a DNA damage response, causing genome instability and cellular senescence [7]. The accumulation of senescent cells impairs tissue function, thereby contributing to the onset of age-related diseases [8]. Numerous meta-analyses show that individuals diagnosed with psychiatric disorders have shorter telomere length (TL) compared to the general population. This has been reported for schizophrenia (SCZ) and related disorders [9], Bipolar Disorder (BD) [10], Major Depressive Disorders (MDD) [11], substance use disorders [12], post-traumatic stress disorder (PTSD) [13] and anxiety disorders [14]. Moreover, a meta-analysis of 32 studies found shortened TL across all psychiatric disorders [15], with the largest effect sizes being observed for PTSD, followed by MDD and anxiety disorders, then by BD and psychotic disorders. Despite this large evidence of TL shortening in individuals with psychiatric disorders, the underlying molecular mechanisms remain unknown.

Telomerase is a holoenzyme located in the nucleus that elongates the end of telomeres when they become critically short. Telomerase reverse transcriptase (TERT) is the telomerase enzymatic subunit that plays an essential role in its activity [16]. TL and telomerase levels in cells gradually decrease with age [17]. Cells deficient in telomerase display a more rapid reduction in TL and exhibit greater DNA damage compared to those with normal telomerase levels [18]. Telomerase has been widely studied in telomere biology disorders where mutations in the *TERT* gene are known to impair telomerase function, leading to shorter TL [18]. In psychiatric research, TL shortening has been hypothesized to be caused by multiple mechanisms. The main mechanism could be an increased telomere attrition due to an excess of oxidative stress [19]. This phenomenon might be worsened by reduced telomere repair due to down-regulation of the *TERT* gene expression, which impairs telomerase activity (TA), thus contributing to TL shortening.

To explore the hypothesis of a decreased TA and/or a down regulation of *TERT* gene expression in psychiatric disorders, we conducted a systematic review of the literature and a meta-analysis with the following aims: 1) to review the case–control studies reporting peripheral blood telomerase activity or *TERT* gene expression in psychiatric disorders, 2) to undertake pooled analyses to examine the magnitude of any differences in telomerase activity or *TERT* gene expression between individuals with psychiatric disorders and controls, 3) to use meta-regression analyses to identify any potential confounders (i.e. factors or variables associated with differences between cases and controls for the reported measures).

## Materials and methods

**Figure 1.**
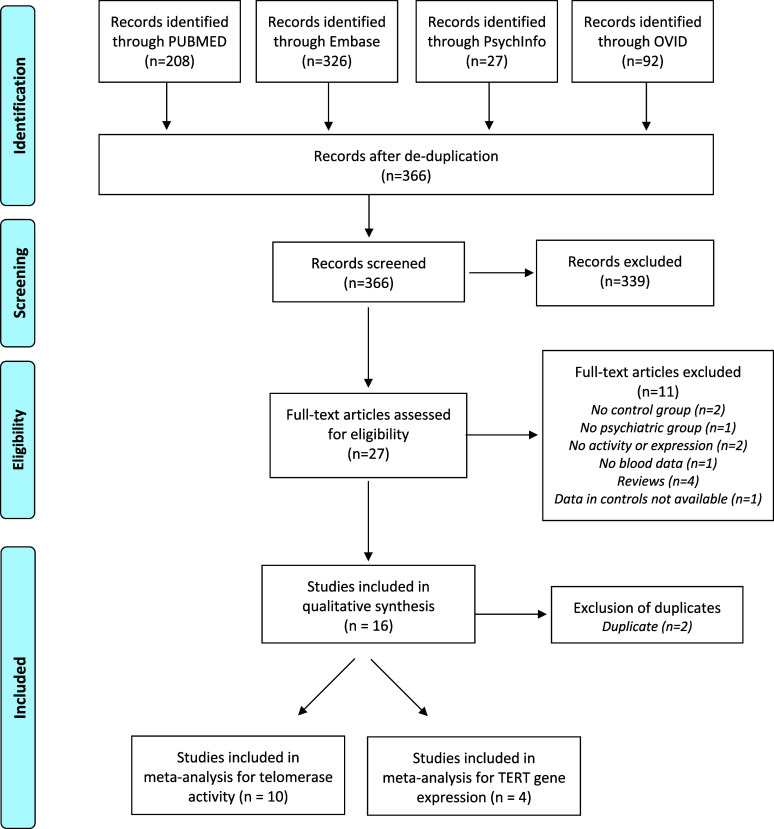
Flow chart of the inclusion and exclusion of studies for the systematic review and the meta-analysis.

The study protocol and the planned meta-analysis were registered in Prospero in October 2024 (reference: CRD42024599331). The project adheres to the Preferred Reporting Items for Systematic Reviews and Meta-analyses (PRISMA) recommendations. A PRISMA checklist is included in Supplementary Appendix 1 and Figure 1 provides a PRISMA flowchart [20].

### Search strategy

A systematic strategy was employed, with electronic databases (MEDLINE, EMBASE, PsycINFO, PubMed, and OVID) searched from inception until November 30, 2024. The following MESH terms for psychiatric disorders were used: (bipolar disorder OR mania OR manic-depress* OR affective psychosis OR depression OR unipolar disorder OR psychosis OR schizophrenia OR anxiety OR substance use OR eating disorders OR PTSD OR ADHD (attention deficit hyperactivity disorder)) AND (telomerase activity OR telomerase expression OR TERT expression). The search was repeated using the following MESH terms for anxiety disorders: (panic disorder OR obsessive-compulsive disorder OR phobia OR social phobia OR generalized anxiety disorder) AND (telomerase activity OR telomerase expression OR TERT expression). The search was repeated using the following MESH terms for substance use disorders: (cannabis OR cocaine OR heroin OR psychostimulant OR alcohol) AND (telomerase activity OR telomerase expression OR TERT expression). The search was repeated using the following MESH terms for eating disorders: (anorexia OR bulimia) AND (telomerase activity OR telomerase expression OR TERT expression). Additionally, we investigated journals that have published articles on related topics, conference proceedings, and citations listed in previous review articles and published studies (that are identified through the database searches). Citation lists were examined, and original investigators were contacted if required to ask for raw or additional data.

### Selection criteria

Publications were assessed for eligibility for the narrative review and then for the meta-analysis.

***Eligibility criteria for the systematic review were*:**

Inclusion:

- English language peer-reviewed articles that reported findings from a case–control study of peripheral blood telomerase activity or *TERT* gene expression,
- cases met internationally recognized criteria for a clinical diagnosis of psychiatric disorders whatever the psychiatric disorder was (e.g. DSM or ICD criteria or agreed clinical consensus),
- comparator groups comprised healthy controls (HC), individuals without a current and/or lifetime history of psychiatric disorders, and/or were reported to be mentally healthy (e.g. they screened negative for major mental disorders),
- telomerase activity and *TERT* gene expression were measured in peripheral blood using laboratory measures with established evidence of reliability and validity.

Exclusion:

- articles that failed to report sample mean scores and standard deviations for the measures of telomerase activity or *TERT* gene expression and/or these data could not be estimated in figures and/or were unavailable from the original investigators,
- articles that reported any of the measures in brain regions.

***Eligibility criteria for the meta-analysis were*:**

- met all the inclusion and none of the exclusion criteria for the systematic review,
- where the original study sample included a range of diagnoses (e.g. BD, schizo-affective disorder, affective psychosis, etc.), the measures in each diagnostic subgroup should be reported or available from the original investigators,
- where more than one publication arose from one dataset (i.e. overlapping datasets), then measures of *TERT* gene expression or telomerase activity would be reported for the largest or the most recent study sample only.

### Procedure

#### Data extraction

We first excluded duplicate publications identified through different databases. Two authors (JT and BE) independently screened the remaining titles for potential eligibility. Some articles were excluded at this stage, while others were excluded after review of the abstracts (see Figure 1). Eligible publications were assessed independently by two authors (JT and BE) who extracted core data regarding sample characteristics (diagnosis, mean age, and gender distribution, size of groups), outcomes (telomerase activity or *TERT* gene expression), and laboratory measures used for the outcomes. Any discrepancies in data extraction were corrected by consensus (JT and BE). Original researchers were contacted as required (e.g. to clarify the independence of samples included in data publications or to obtain additional data) and articles were excluded if clarifications or data were unavailable or there was no response.

#### Risk of bias (quality) assessment

Quality of included studies was assessed using the Newcastle-Ottawa Quality Assessment Scale for Case Control Studies (NOS). Assessors reviewed and critically appraised each publication independently (total score ranged from 0 to 7) and then recorded a jointly agreed score and quality rating.

### Data reporting and statistical analyses

#### Systematic review

Main characteristics of the samples and main findings from studies were summarized in tabular form and with a written description.

#### Meta-analyses

We extracted data for means and standard deviations (SD) for each measure (telomerase activity and *TERT* gene expression). If only medians and inter-quartile ranges were reported, these were transformed into means and SDs using Metaconverter. If data were available only in figures (i.e. without raw data in table or text), we contacted original researchers or used the software Image J to extract the data.

Analyses of pooled data from eligible studies were undertaken using SPSS and the R ‘METAFOR’ package (Meta-Analysis Package for R) [21]. We undertook a series of pooled analyses (e.g. all studies eligible for meta-analysis for each given outcome) when at least three independent studies reported data for the same outcome. Random effects modelling for pooled effect sizes (ES) were employed [22]. The standardized mean difference (SMD) and 95% confidence intervals (95% CI) were estimated for each study. The SMD was defined as the difference in means between the two groups (cases versus controls) divided by the pooled standard deviation of the measures and was interpreted in a similar manner to Cohen’s *d* (0.2 = small ES; 0.5 = medium ES; 0.8 = large ES).

We used SPSS to construct forest plots, while publication bias was assessed by visual inspection of the funnel plots and tested using the rank correlation test for funnel plot asymmetry. The *I*^2^ statistic was used to quantify heterogeneity, with the values of 25, 50 and 75% reflecting a small, moderate, or high degree of heterogeneity, respectively [23]. Also, in line with recent suggestions for reporting, we include Cochran’s Q statistics and the corresponding *p* values.

#### Meta-regression analyses

Meta-regression analyses were performed using the R METAFOR package, which performs random-effects meta-regression using aggregate-level data. This function uses an iterative method to produce estimates (Beta and SDs) and *p*-values. Meta-regressions were undertaken for: year of publication, study total sample size, diagnosis (mood disorders vs other psychiatric disorders), age (difference between mean age of cases vs. controls), sex distribution (ratio of percentages of females in cases and controls), cell types (peripheral blood mononuclear cells vs other peripheral blood cells), and study quality.

## Results

### Literature search

The literature search produced a set of 653 articles (see Figure 1). After de-duplication, the set was reduced to 366 articles. Following review of titles and abstracts, this decreased further to 27 articles, and after full-text assessments, 16 publications met eligibility criteria for the qualitative synthesis. From those, 14 studies were found to be eligible for the meta-analyses, with 10 for telomerase activity [24–30] (Wolkowitz unpublished) and 2 studies were identified as duplicates [31, 32]. The remaining 4 studies were eligible for *TERT* gene expression [33–36].

### Systematic review

Details of each included study are presented in Table 1 and Table 2. Articles were published between 2008–2024, with most (*n* = 10/16) appearing in the last decade. Only one study in PTSD has not yet been formally published, that is data were reported only as *p* values in the discussion of the original article [38], but investigators agreed to provide the raw data (study further labelled as “Wolkowitz et al. unpublished). These partially published data were considered eligible for the systematic review and the meta-analysis. Most samples (*n* = 7/16) were from the USA, followed by Europe (*n* = 6/16). Sample sizes were small, with most studies including less than 100 cases and 100 controls. Only two studies had large sample sizes: Simon et al. [26] with 166 individuals with MDD and 166 controls (telomerase activity) and Mlakar et al. [36] with 357 individuals with SCZ and 401 controls (*TERT* gene expression). Studies mainly included individuals diagnosed with MDD (*n* = 8/16). All control groups had been screened to confirm the absence of psychiatric disorders. One study [28] included a mixed control group consisting of healthy controls and unaffected relatives of cases. Most studies included both females and males, except for five studies: 2 in PTSD [30] (Wolkowitz et al. unpublished), 1 in BD [24] and 1 in heroin use disorder that included only males [29] and 1 in MDD that included only females [33]. The matching for gender was correct for all studies except one [24]. The matching for age was correct for most studies except one [28]. Twelve studies investigated telomerase activity, while only four investigated *TERT* gene expression. Regarding telomerase activity, all studies used a similar method, i.e. TRAP (Telomere Repeat Amplification Protocol). Regarding *TERT* gene expression, 3 out of 4 studies used RT-qPCR, while one used microarrays [36]. Overall, the study quality score was good with 8/16 studies having a score of 6 or 7 (score can range from 0 to 7).

**Table 1.** Telomerase activity

Authors	Year	Country	Patients, N	Controls, N	Diagnosis criteria	Mean age	Proportion of females	Sampling source	Method	Main results	Remarks
**Eligible studies**											
Soeiro-de-Souza et al.	2014 [24]	Brazil	28 patients with BD	23 without lifetime history of any axis I psychiatric disorder	DSM-IV	Patients = 28 yo controls = 27 yo	Patients = 75% controls = 43%	PBMC	TRAP	BD = HC *p* = 0.64	
Wolkowitz et al.	2015 [25]	USA	25 patients with MDD	18 subjects without present or history of any DSM-IV Axis I diagnosis	DSM-IV	Patients = 38 yo controls = 35 yo	Patients = 68% controls = 61%	PBMC	TRAP	MDD > HC *p* = 0.025	
Simon et al.	2015 [26]	USA	166 patients with MDD	166 subjects without any DSM-IV Axis I psychiatric disorder	DSM-IV	Patients = 41 yo controls = 41 yo	Patients = 54% controls = 54%	Leukocytes	TRAP	MDD = HC *p* = 0.40	
Ryan et al.	2021	Ireland	20 patients with MDD	33 healthy controls	DSM-IV	Patients = 58 yo controls = 51 yo	Patients =50% controls = 58%	PBMC	TRAP	MDD = HC *p* = 0.79	
Bürhan-Çavuşoğlu et al.	2021 [37]	Turkey	39 patients with MDD	39 healthy volunteers	DSM-IV	Patients = 33 yo controls = 32 yo	Patients =72% controls =72%	PBMC	TRAP	MDD > HC *p* = 0.001	
Walia et al.	2023 [27]	India	35 patients with MDD (depression)	35 with General Health Questionnaire-12 below 3	DSM-5	Patients = 34 yo controls = 34 yo	Patients = 51% controls = 51%	PBMC	TRAP	MDD > HC *p* < 0.001	
Porton et al.	2008 [28]	USA	53 patients with SZ	Total of 84 (59 unaffected controls +25 unaffected first-degree family members)	DSM-IV	Patients = 38 yo controls = 28 yo	NA	Lymphocytes	TRAP	SZ < HC *p* = 0.01	
Cheng et al.	2013 [29]	China	33 abstinent heroin users	30 without psychiatric disorder	NA	Patients = 35 yo controls =33 yo	Patients = 0% controls = 0%	PBMC	TRAP	Heroin < HC *p* < 0.001	
Jergovic et al.	2014 [30]	Croatia	30 patients with PTSD	14 without history of acute psychosis, dementia, mood disorders, schizophrenia, or personality disorders	ICD-10	Patients = 46 yo controls = 47 yo	Patients = 0% controls = 0%	PBMC	TRAP	PTSD = HC	
Wolkowitz et al.	unpublished	USA	81 patients with PTSD	79 veterans without PTSD nor MDD	DSM-IV	Patients =33 yo controls =33 yo	Patients = 0% controls = 0%	Leukocytes	TRAP	PTSD = HC *p* = 0.76	From Verhoeven et al. 2018 [38]
**Non eligible studies**											
Wolkowitz et al.	2012 [31]	USA	20 patients with MDD	18 subjects without present or past history of any DSM-IV Axis I or Axis II diagnosis	DSM-IV	Patients = 37 yo controls = 35 yo	Patients = 65% controls = 67%	PBMC	TRAP	MDD > HC *p* = 0.007	Duplicate
Chen et al.	2014 [32]	USA	20 patients with MDD	20 without present or past history of any DSM-IV Axis I diagnosis	DSM-IV	NA matched on age +/− 3 years	NA matched on gender	PBMC	TRAP	MDD > HC *p* = 0.016	Duplicate

Abbreviations: MDD: Major Depressive Disorder, PTSD: Post-Traumatic Stress Disorder, BD: Bipolar Disorder, SZ: Schizophrenia; N: Number, DSM: Diagnostic and Statistical Manual of Mental Disorders, ICD: International Classification of Diseases, NA: Not Available, USA: United States of America, yo: year old, TRAP: Telomerase Repeated Amplification Protocol, HC: Healthy Controls, PBMC: Peripheral blood mononuclear cell

**Table 2.** TERT gene expression

Authors	Year	Country	Patients, N	Controls, N	Diagnosis criteria	Mean age	Proportion of females	Sampling source	Method	Main results	Remarks
**Eligible studies**											
Teyssier et al.	2012 [33]	France	17 patients with MDD	16 healthy subjects without Psychiatric diagnostic	DSM-IV	Patients = 39 yo Controls = 38 yo	Patients = 100% Controls = 100%	Leukocytes	RT-qPCR	MDD > HC *p* = 0.05	
Köse Çinar et al.	2018 [34]	Turkey	21 patients with BD (manic)	20 subjects with no history of psychiatric disorder or Medical illness and who had no first-degree relatives with BD, schizophrenia, or other psychotic disorders	DSM-IV	Patients = 31 yo Controls = 32 yo	Patients = 0% Controls = 0%	Peripheral Blood	RT-qPCR	manic > HC *p* = 0.03	
Köse Çinar et al.	2018 [34]	Turkey	21 patients with BD (remission)	20 subjects with no history of psychiatric disorder or Medical illness and who had no first-degree relatives with BD, schizophrenia, or other psychotic disorders	DSM-IV	Patients = 31 yo Controls = 32 yo	Patients = 0% Controls = 0%	Peripheral blood	RT-qPCR	remitted BD > HC *p* = 0.01	
Lundberg et al.	2020 [35]	USA	97 patients with BD 1	100 participants with no history of psychiatric disorder or first-degree relatives with BD and ICD-9 codes associated with BD and/or schizophrenia	DSM-IV and ICD-9	Patients = 45 yo controls = 45 yo	Patients = 65% controls = 65%	Peripheral blood leukocyte	RT-qPCR	BD = HC *p* = 0.40	
Mlakar et al.	2024 [36]	Norway	357 patients with SZ	401 subjects with no current or lifetime diagnosis of a severe mental disorder or substance abuse or dependency, and no severe mental disorders in their close relatives	DSM-IV	Patients = 29 yo controls = 31 yo	Patients = 41% controls = 43%	Peripheral Blood	Microarray	SZ < HC *p* = 0.03	
**Non eligible studies**											
Mlakar et al.	2024 [36]	Norway	357 patients with SZ	401 subjects with no current or lifetime diagnosis of a severe mental disorder or substance abuse or dependency and no severe mental disorders in their close relatives	DSM-IV	Patients = 29 yo controls = 31 yo	Patients = 41% controls = 43%	Peripheral blood	Microarray	SZ = HC *p* = 0.31	TERC expression

Abbreviations: MDD: major depressive disorder, BD: bipolar disorder, SZ: schizophrenia; *N*: number, DSM: diagnostic and Statistical Manual of Mental Disorders, ICD: International Classification of Diseases, NA: not available, USA: United States of America, yo: year old, RT-qPCR: quantitative reverse transcription polymerase chain reaction, HC: healthy controls, PBMC: peripheral blood mononuclear cell, TERT: telomerase reverse transcriptase, TERC: telomerase RNA component.

### Meta-analysis

We undertook two separate meta-analyses, one for telomerase activity and one for *TERT* gene expression.

#### Telomerase activity

**Figure 2.**
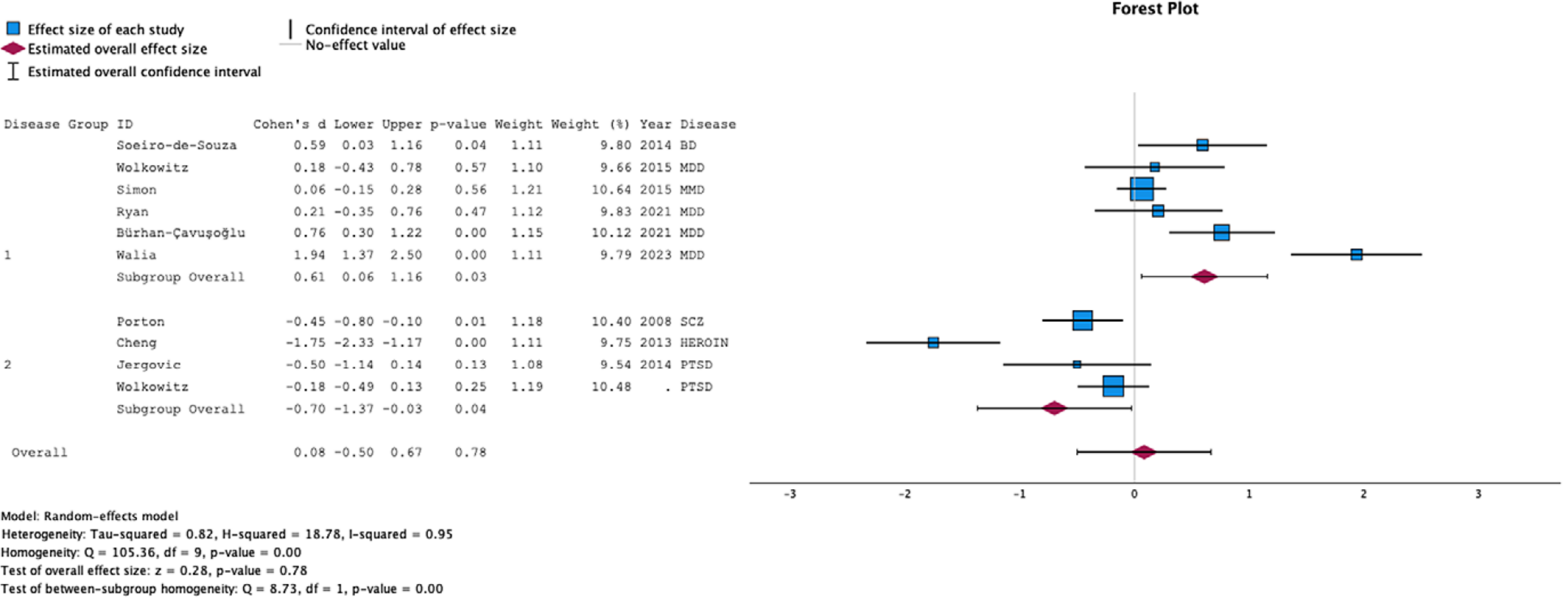
Forest plot of cases versus controls comparison for telomerase activity. ID: name of first author of the article, BD: Bipolar Disorder, Heroin: Heroin use disorder, MDD: Major Depressive Disorder, PTSD: Post-Traumatic Stress Disorder, SZ: Schizophrenia.

The meta-analysis was based on 10 studies, including a total of 510 cases and 521 controls. We stratified the analyses on the type of psychiatric disorders (individuals with MDD and depressed individuals with BD versus other psychiatric disorders). As shown in Figure 2, when all psychiatric disorders were considered, the pooled ES was 0.08 [−0.50–0.67], *p* = 0.78. A significant heterogeneity was detected between studies (I-squared = 0.95, *p* < 0.001). Funnel plots are provided in Supplementary Figure S1. Rank correlation tests for funnel plot asymmetry did not reveal evidence of publication bias (Kendall’s tau = −0.02 *p* = 1.00).

Analyses stratified on the types of psychiatric disorders (mood disorders versus others) yielded an ES of 0.61 [0.06–1.16] (*p* = 0.03) for mood disorders and an ES of −0.70 [−1.37 to −0.03] (*p* = 0.04) for other psychiatric disorders.

According to meta-regression analyses, larger ES were observed in studies involving individuals with mood disorders versus other types of psychiatric disorders (*p* = 0.003), but also in more recent studies (*p* = 0.01). Total number of included participants (*p* = 0.86), case–control gender or age differences (respectively *p* = 0.67 and *p* = 0.56), analysis in PBMCs rather than other peripheral blood cells (*p* = 0.55) and quality of the studies (*p* = 0.13) did not influence ES. Meta-regression plots are presented for the year of publication and types of psychiatric disorders in Supplementary Figures S2 and S3.

#### *TERT* gene expression

**Figure 3.**
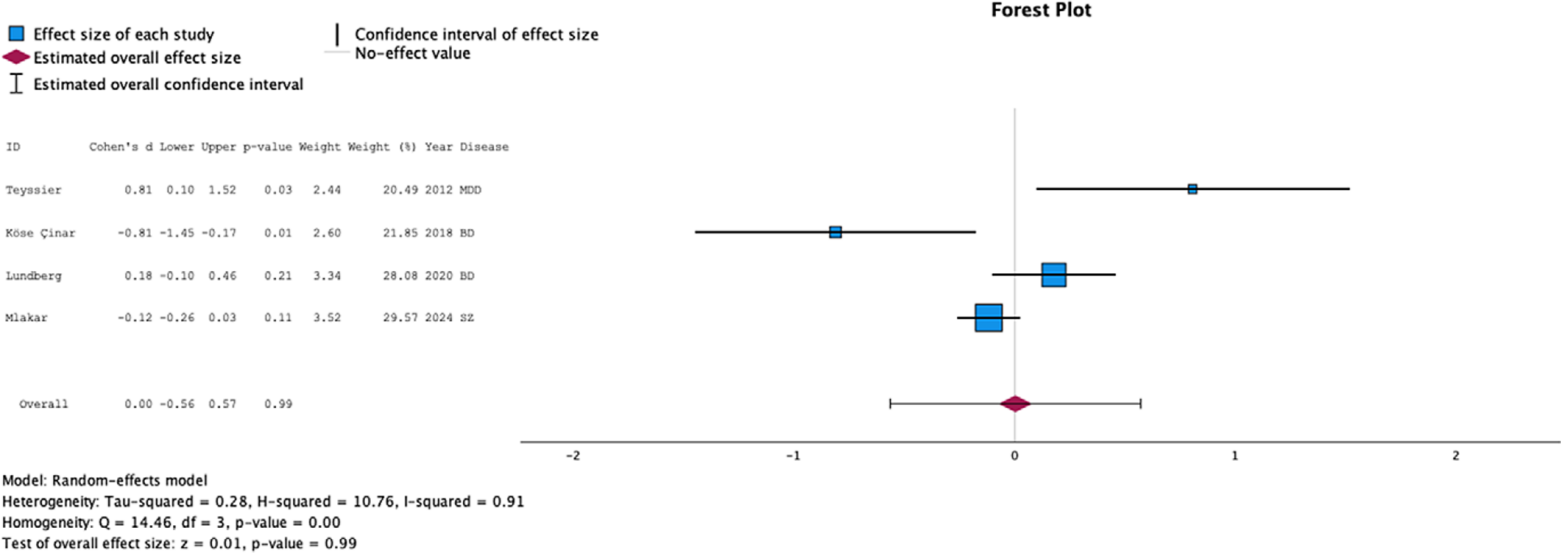
Forest plot of cases versus controls comparison for telomerase gene expression. ID: name of first author of the article, BD: Bipolar Disorder, MDD: Major Depressive Disorder, SZ: Schizophrenia. For Köse Çinar et al. 2018, only data for BD cases in remission versus controls are presented.

The meta-analysis was based on four studies, including a total of 492 cases and 537 controls. Since one study included the same cases versus controls [34] – but at different time points (during mania then in remission), we first performed a meta-analysis including only the data for the individuals in remission versus controls. As shown in Figure 3, the pooled ES was 0.00 [−0.56–0.57], *p* = 0.99. A significant heterogeneity was detected between studies (I-squared = 0.91, *p* < 0.001). Funnel plots are provided in Supplementary Figure S4. Rank correlation tests for funnel plot asymmetry did not reveal any evidence of publication bias (Kendall’s tau = 0.33 *p* = 0.75).

The meta-analyses were repeated, including only the data from the same study [34] but for the individuals during mania versus controls. This analysis led to similar results with an ES of 0.03 [−0.47–0.53] (*p* = 0.91) (data not shown in detail).

No meta-regression analysis for *TERT* gene expression was performed, given the small number of studies.

## Discussion

Telomere shortening is a phenomenon shared by most psychiatric disorders, leading to the hypothesis of alterations of telomerase activity (TA) and/or *TERT* expression. To the best of our knowledge, this is the first meta-analysis that investigates the association between peripheral blood telomerase activity and *TERT* gene expression levels across psychiatric disorders compared with healthy controls. We showed that peripheral blood TA and *TERT* gene expression do not differ between individuals with psychiatric disorders compared to healthy controls. However, stratification of the analyses shows an increase of TA in mood disorders (MDD and BD), and a decrease of TA in non-mood disorders (PTSD, SCZ, heroin use disorder).

We identified important limitations in our systematic review that might explain the results when considering all psychiatric disorders. First, the search retrieved a limited number of studies, with some duplicates or overlapping samples for TA and even fewer studies for *TERT* expression. Second, most studies – except for two – included small sample sizes. These two observations may have limited the ability to detect any differences between cases and controls. Third, all (except one) studies used a cross-sectional design, meaning that the data were collected at a given point in time. This limitation prevents a deeper understanding of how *TERT* gene expression and TA may fluctuate over time or depend on symptom levels, but also prevents observing their variations over the progression of chronic disorders.

Of particular interest is the difference in effect sizes for telomerase activity between non-mood disorders and mood disorders. In non-mood disorders, the hypothesis of an underlying mechanism linking shorter telomere length to lower telomerase activity (TA) may be valid, since TA was decreased in these conditions. However, this observation may be driven by the study in heroin-abstinent individuals [29], which reported the lowest effect size. For instance, it has been shown that chronic stress can lead to a secondary adaptation that suppresses *TERT* expression and TA [39], which could be the case for non-mood disorders. Furthermore, in a recent longitudinal study, chronic stress predicts lower Mitochondrial Health Index (a composite marker integrating mitochondrial energy-transformation capacity and content) that in turn predicts decreases in telomerase activity and lower TL [40].

For mood disorders, the results are in the opposite direction, that is increased TA both in MDD and BD (all samples were collected during a depressive episode) as compared to controls. Studies in MDD mostly include drug-naïve individuals exhibiting severe depressive symptoms at inclusion. Based on the previously mentioned mechanistic hypothesis, these individuals would be expected to demonstrate low TA levels. Our findings are therefore counter intuitive. Equally unexpected is the observation that some of these studies reported a positive correlation between TA and the severity of depressive symptoms [27, 31, 37]. Since most studies (except one) are cross-sectional, a one-point measure may reflect or overlook a secondary adaptation. For example, acute stress can induce telomere attrition by increasing reactive oxygen species (ROS), which in turn leads to elevated *TERT* expression and TA, which might be the case during a depressive episode. However, when stress becomes chronic, a secondary adaptation suppresses *TERT* expression and TA [39] which might explain the opposite observation in non-mood disorders.

The difference observed for TA in mood and non-mood disorders may reflect differences in treatment regimens. Indeed, most studies examining mood disorders included drug-naïve participants. In studies investigating TA in non-mood disorders, medication regimens were heterogenous (not reported, naive patients, or treated patients). Therefore, no meta-regression analysis was feasible to assess the impact of the different pharmacological interventions on TA. In a recently published review, we have shown that very few studies assessed the effect of psychiatric medications on TA in longitudinal designs [41]. We identified only two studies in MDD, both showing no significant change of TA pre-vs post-antidepressants, and only one study in BD showing no significant change of TA pre-vs post-lithium. Therefore, future studies should accurately describe the medication regimens at inclusion and further explore any potential associations with TA.

Most importantly, the maintenance of telomeres is a complex biological mechanism and the sole study of TA or *TERT* protein may be too restrictive. The three primary multiprotein complexes central to this process are the Shelterin complex, the CST complex, and the telomerase complex (with five other proteins than TERT and one non-coding RNA), along with numerous accessory proteins. In telomere biology disorders, research has identified 16 genes encoding proteins that regulate telomere homeostasis. Alterations in any of these genes can lead to excessive telomere shortening and dysfunction [42]. Supporting the hypothesis that other proteins than TERT may play a role in TL shortening in psychiatric disorders, previous studies have identified two of these proteins as potentially implicated. One study identified a downregulation of the CST protein *CTC1* in individuals with schizophrenia as compared to controls through whole-genome expression profiling [43]. Another study analyzed the expression of 29 genes involved in telomere homeostasis and aging and observed a downregulation of *POT1* in individuals with bipolar disorder with shorter telomeres, compared to age-matched cases with normal telomere length [44]. As a constituent of the Shelterin complex, *POT1* plays a pivotal role in the recruitment of telomerase and the CST complex, both of which are critical for the elongation of telomeres [45, 46]. These two studies provide new insights into the understanding of TL in psychiatric disorders that go beyond the sole investigation of TA or *TERT* gene expression [41].

## Conclusion

This meta-analysis shows that psychiatric disorders – when considered independently of diagnoses – are not associated with peripheral blood telomerase activity nor *TERT* gene expression. However, we suggest that telomerase activity is increased in depressive unipolar or bipolar disorders, while it is decreased in non-mood psychiatric disorders. The paucity of studies and small sample sizes are important limitations of the available literature, particularly for *TERT* expression. Future studies are therefore required, which would include larger samples and a wider range of psychiatric disorders. Although telomerase and its catalytic subunit TERT have been suggested to play a central role in TL shortening in psychiatric disorders, more comprehensive investigations of other biological pathways involved in telomere homeostasis are essential to understand the mechanisms at stake.

## Supporting information

10.1192/j.eurpsy.2025.10099.sm001 Thurin et al. supplementary material Thurin et al. supplementary material

## Data Availability

Data extracted from included studies and data used for all analyses are available upon request to the authors.

## Supplementary material

The supplementary material for this article can be found at http://doi.org/10.1192/j.eurpsy.2025.10099.
