# Supplementary material for: Peripheral blood telomerase activity and telomerase reverse transcriptase gene expression in psychiatric disorders: a systematic review and meta-analysis
Source: Eur Psychiatry. 2025 Sep 1;68(1):e123. doi: 10.1192/j.eurpsy.2025.10099 (PMC12438989; doi:10.1192/j.eurpsy.2025.10099)
Supplement: Thurin et al. supplementary material [file S0924933825100990sup001.docx]

Table 1:

TELOMERASE ACTIVITY

| **Authors** | **Year** | **Country** | **Patients, N** | **Controls, N** | **Diagnosis criteria** | **Mean age** | **Proportion**  **of females** | **Sampling source** | **Method** | **Main results** | **Remarks** |
| --- | --- | --- | --- | --- | --- | --- | --- | --- | --- | --- | --- |
| **Eligible studies** | | | | | | | | | | | |
| Soeiro-de-Souza et al. | 2014 | Brazil | 28 patients  with BD | 23 without lifetime history of any axis I psychiatric disorder | DSM-IV | patients = 28 yo  controls = 27 yo | patients = 75%  controls = 43% | PBMC | TRAP | BD = HC  p = 0.64 |  |
| Wolkowitz et al. | 2015 | USA | 25 patients  with MDD | 18 subjects without present or history of any DSM-IV Axis I diagnosis | DSM-IV | patients = 38 yo  controls = 35 yo | patients = 68%  controls = 61% | PBMC | TRAP | MDD > HC  p = 0.025 |  |
| Simon et al. | 2015 | USA | 166 patients with MDD | 166 subjects without any DSM-IV Axis I psychiatric disorder | DSM-IV | patients = 41 yo  controls = 41 yo | patients = 54%  controls = 54% | Leukocytes | TRAP | MDD = HC  p = 0.40 |  |
| Ryan et al. | 2021 | Ireland | 20 patients  with MDD | 33 healthy controls | DSM-IV | patients = 58 yo  controls = 51 yo | patients =50%  controls = 58% | PBMC | TRAP | MDD = HC  p = 0.79 |  |
| Bürhan-Çavuşoğlu et al. | 2021 | Turkey | 39 patients with MDD | 39 healthy volunteers | DSM-IV | patients = 33 yo  controls = 32 yo | patients =72%  controls =72% | PBMC | TRAP | MDD > HC  p = 0.001 |  |
| Walia et al. | 2023 | India | 35 patients  with MDD (depression) | 35 with General Health Questionnaire-12 below 3 | DSM-5 | patients = 34 yo  controls = 34 yo | patients = 51%  controls = 51% | PBMC | TRAP | MDD > HC  p < 0.001 |  |
| Porton et al. | 2008 | USA | 53 patients  with SZ | Total of 84 (59 unaffected controls + 25 unaffected first-degree family members) | DSM-IV | patients = 38 yo  controls = 28 yo | NA | Lymphocytes | TRAP | SZ < HC  p = 0.01 |  |
| Cheng et al. | 2013 | China | 33 abstinent heroin users | 30 without psychiatric disorder | NA | patients = 35 yo  controls =33 yo | patients = 0%  controls = 0% | PBMC | TRAP | heroin < HC  p < 0.001 |  |
| Jergovic et al. | 2014 | Croatia | 30 patients  with PTSD | 14 without history of acute psychosis, dementia, mood disorders, schizophrenia, or personality disorders | ICD-10 | patients = 46 yo  controls = 47 yo | patients = 0%  controls = 0% | PBMC | TRAP | PTSD = HC |  |
| Wolkowitz et al. | unpublished | USA | 81 patients  with PTSD | 79 veterans without PTSD nor MDD | DSM-IV | patients =33 yo  controls =33 yo | patients = 0%  controls = 0% | Leukocytes | TRAP | PTSD = HC  p = 0.76 | from Verhoeven et al. 2018 |
| **Non eligible studies** | | | | | | | | | | | |
| Wolkowitz et al. | 2012 | USA | 20 patients  with MDD | 18 subjects without present or past  history of any DSM-IV Axis I or Axis II diagnosis | DSM-IV | patients = 37 yo  controls = 35 yo | patients = 65%  controls = 67% | PBMC | TRAP | MDD > HC  p = 0.007 | duplicate |
| Chen et al. | 2014 | USA | 20 patients  with MDD | 20 without present or past  history of any DSM-IV Axis I diagnosis | DSM-IV | NA  matched on age  +/- 3 years | NA  matched on gender | PBMC | TRAP | MDD > HC  p = 0.016 | duplicate |

MDD: Major Depressive Disorder, PTSD: Post-Traumatic Stress Disorder, BD: Bipolar Disorder, SZ: Schizophrenia

N: Number, DSM: Diagnostic and Statistical Manual of Mental Disorders, ICD: International Classification of Diseases, NA: Not Available, USA: United States of America, yo: year old, TRAP: Telomerase Repeated Amplification Protocol, HC: Healthy Controls, PBMC: Peripheral blood mononuclear cell

TERT GENE EXPRESSION

| **Authors** | **Year** | **Country** | **Patients, N** | **Controls, N** | **Diagnosis criteria** | **Mean age** | **Proportion**  **of females** | **Sampling source** | **Method** | **Main results** | **Remarks** |
| --- | --- | --- | --- | --- | --- | --- | --- | --- | --- | --- | --- |
| **Eligible studies** | | | | | | | | | | | |
| Teyssier et al. | 2012 | France | 17 patients  with MDD | 16 healthy subjects without  psychiatric diagnostic | DSM-IV | patients = 39 yo  controls = 38 yo | patients = 100%  controls = 100% | leukocytes | RT-qPCR | MDD > HC  p= 0.05 |  |
| Köse Çinar et al. | 2018 | Turkey | 21 patients with BD (manic) | 20 subjects with no history of psychiatric disorder or  medical illness and who had no first-degree relatives with  BD, schizophrenia, or other psychotic disorders | DSM-IV | patients = 31 yo  controls = 32 yo | patients = 0%  controls = 0% | Peripheral Blood | RT-qPCR | manic > HC  p = 0.03 |  |
| Köse Çinar et al. | 2018 | Turkey | 21 patients with BD (remission) | 20 subjects with no history of psychiatric disorder or  medical illness and who had no first-degree relatives with  BD, schizophrenia, or other psychotic disorders | DSM-IV | patients = 31 yo  controls = 32 yo | patients = 0%  controls = 0% | Peripheral Blood | RT-qPCR | remitted BD > HC  p = 0.01 |  |
| Lundberg et al. | 2020 | USA | 97 patients  with BD 1 | 100 participants with no history of psychiatric disorder or first-degree relatives with BD and ICD-9 codes associated with BD and/or schizophrenia | DSM-IV and ICD-9 | patients = 45 yo  controls = 45 yo | patients = 65%  controls = 65% | Peripheral blood leukocyte | RT-qPCR | BD = HC  p = 0.40 |  |
|  |  |  |  |  |  |  |  |  |  |  |  |
| Mlakar et al. | 2024 | Norway | 357 patients with SZ | 401 subjects with no current or lifetime diagnosis of a severe mental disorder or substance abuse or dependency and no severe mental disorders in their close relatives | DSM-IV | patients = 29 yo  controls = 31 yo | patients = 41%  controls = 43% | Peripheral Blood | microarray | SZ < HC  p = 0.03 |  |
| **Non eligible studies** | | | | | | | | | | | |
| Mlakar et al. | 2024 | Norway | 357 patients with SZ | 401 subjects with no current or lifetime diagnosis of a severe mental disorder or substance abuse or dependency and no severe mental disorders in their close relatives | DSM-IV | patients = 29 yo  controls = 31 yo | patients = 41%  controls = 43% | Peripheral Blood | microarray | SZ = HC  p = 0.31 | TERC expression |

MDD: Major Depressive Disorder, BD: Bipolar Disorder, SZ: Schizophrenia

N: Number, DSM: Diagnostic and Statistical Manual of Mental Disorders, ICD: International Classification of Diseases, NA: Not Available, USA: United States of America, yo: year old, RT-qPCR: Quantitative reverse transcription polymerase chain reaction, HC: Healthy Controls, PBMC: Peripheral blood mononuclear cell

TERT: telomerase reverse transcriptase, TERC: Telomerase RNA component
